# Risk Stratification of Sentinel Node Metastasis Disease Burden and Phenotype in Stage III Melanoma Patients

**DOI:** 10.1245/s10434-022-12804-6

**Published:** 2022-11-29

**Authors:** Zahra Hussain, Martin J. Heaton, Andrew P. Snelling, Jenny P. Nobes, Gill Gray, Jennifer J. Garioch, Marc D. Moncrieff

**Affiliations:** 1 Department of Plastic Surgery and Reconstructive Surgery, Norfolk and Norwich University Hospital, Norwich, UK; 2 Department of Oncology, Norfolk and Norwich University Hospital, Norwich, UK; 3 Norwich Medical School, University of East Anglia, Norwich, UK; 4 Department of Dermatology, Norfolk and Norwich University Hospital, Norwich, UK

## Abstract

**Background:**

Currently, all patients with American Joint Committee on Cancer (AJCC) pT2b-pT4b melanomas and a positive sentinel node biopsy are now considered for adjuvant systemic therapy without consideration of the burden of disease in the metastatic nodes.

**Methods:**

This was a retrospective cohort analysis of 1377 pT1-pT4b melanoma patients treated at an academic cancer center. Standard variables regarding patient, primary tumor, and sentinel node characteristics, in addition to sentinel node metastasis maximum tumor deposit size (MTDS) in millimeters and extracapsular spread (ECS) status, were analyzed for predicting disease-specific survival (DSS).

**Results:**

The incidence of SN+ was 17.3% (238/1377) and ECS was 10.5% (25/238). Increasing AJCC N stage was associated with worse DSS. There was no difference in DSS between the IIIB and IIIC groups. Subgroup analyses showed that the optimal MTDS cut-point was 0.7 mm for the pT1b-pT4a SN+ subgroups, but there was no cut-point for the pT4b SN+ subgroup. Patients with MTDS <0.7 mm and no ECS had similar survival outcomes as the N0 patients with the same T stage. Nodal risk categories were developed using the 0.7 mm MTDS cut-point and ECS status. The incidence of low-risk disease, according to the new nodal risk model, was 22.3% (53/238) in the stage III cohort, with 49% (26/53) in the pT2b-pT3a and pT3b-pT4a subgroups and none in the pT4b subgroup. Similar outcomes were observed for overall and distant metastasis-free survival.

**Conclusion:**

We propose a more granular classification system, based on tumor burden and ECS status in the sentinel node, that identifies low-risk patients in the AJCC IIIB and IIIC subgroups who may otherwise be observed.

**Supplementary Information:**

The online version contains supplementary material available at 10.1245/s10434-022-12804-6.

The classification for stage III melanoma, namely patients with in-transit metastases and/or regional nodal metastases, is complex and reflects the diverse prognostic outcomes of this heterogeneous group of patients.^1^ The addition of sentinel node (SN) biopsy (SNB) in the staging pathway for high-risk primary melanoma patients affords a further level of complexity by identifying nodal metastases early in their evolution at the micrometastatic stage, while the diagnostic test, for the majority of patients, simultaneously removes the metastatic focus in the lymph nodes.^2,3^ Furthermore, there are the competing risks of developing distant metastases from either the primary tumor and/or the locoregional metastases. In an attempt to stratify these layers of complexity into a simplified risk spectrum for guiding subsequent treatment, the latest iteration of the American Joint Committee on Cancer (AJCC) classification system (8th edition)^1^ of stage III melanoma incorporates not only prognostic characteristics of the locoregional spread but also characteristics of the original primary, and combines them into four prognostic levels, namely IIIA–IIID. At one end of this spectrum are those with early-stage melanoma primaries (pT1b-pT2a; AJCC 8th edition) who have low disease burden micrometastases detected at SNB. These patients are mapped to the AJCC IIIA subcategory. Nearly all patients with AJCC IIIA melanoma, representing low disease-burden, low-risk stage III disease, have an excellent prognosis, with 5-year survival approaching 90%.^1^ Recently, a multinational consortium identified a subgroup of putatively low-risk AJCC IIIA patients with a greatly increased risk of recurrence and/or death, based on disease burden in the SN, measured as the maximum tumor deposit size (MTDS).^4^

At the other end of this spectrum, the indications for offering adjuvant systemic therapy to patients with intermediate- to high-risk AJCC stage III, namely IIIB–IIID, melanoma have largely been standardized internationally.^5,6^ Accordingly, all patients with pT2b-pT4b melanomas and a positive SNB are now considered for adjuvant systemic therapy without consideration of the burden of disease in the metastatic nodes, which could potentially have a bearing on conversations with patients regarding the benefits from treatment and the risks from toxicity and overtreatment. This study sought to identify the incidence of patients with intermediate/high-risk melanoma primaries (AJCC pT2b-pT4b) who had micrometastases diagnosed at SNB, and to determine the prognostic information associated with spectrum of disease burden in the metastatic nodes. The aim was to stratify patients with putatively high-risk SN+ AJCC IIIB–IIID into those who are more likely to benefit from adjuvant systemic therapy and those who could potentially be observed.

## Methods

Central regulatory approval for this study was granted by the UK National Health Service (NHS) Health Research Authority (IRAS ID: 297203). This was a single-center, retrospective cohort analysis using data collated from a prospective, institutionally maintained melanoma database at an academic cancer center. The primary inclusion criteria were adult patients (>18 years of age) with pathological stage pT1b-pT4b primary cutaneous melanomas (AJCC 8th edition classification) who underwent SNB between 2008 and 2020. Patients with microsatellites were excluded from this analysis.

Standard patient demographic data and tumor characteristics were recorded. Mitotic rate, defined as mitoses per square millimeter, was measured as per the AJCC criteria using the ‘hot-spot’ method. Details of the SNB report included nodal status, and, for the SN+ cases, N stage, maximum size of tumor deposit, and presence of extracapsular spread (ECS; synonyms: extracapsular extension, extranodal spread; extranodal extension). Historic completion lymph node dissection (CLND) data were not used in the analysis due to the bias of many SN+ patients participating in the MSLT-2 study at the same time. Similarly, patients were not stratified according to adjuvant systemic therapy since this was only standard of care from late 2018.

Survival outcomes data were collected, including disease-specific survival (DSS), distant metastasis-free survival (DMFS), and overall survival (OS) according to standard US FDA criteria.^7^ In the case of multiple sites of recurrence, disease-free survival (DFS) was recorded based on the first instance and highest stage at that time, according to the ‘first/worst’ principle.

### Statistical Analysis

Pseudoanonymized data were analyzed using Jamovi software (version 1.6; Sydney, NSW, Australia; https://www.jamovi.org) and R-Studio (version 1.3.1093; Boston, MA, USA), both running R language (version 3.6; https://cran.r-project.org/). Patient characteristics and histopathological parameters were summarized using descriptive statistics stratified by SN status. Medians with interquartile ranges (IQR) and frequency with proportion were used for continuous and categorical variables, respectively. Differences in the median were tested using Kruskal–Wallis tests for continuous variables, and Chi-square tests were used to assess differences in proportions. All survival outcomes were summarized using the Kaplan–Meier method, and differences between groups were assessed using the log-rank test.

The functional relationship between the maximum SN metastatic deposit size and survival outcomes was analyzed to identify an optimal cut-point that separates low- and high-risk groups within stage III patients with early-stage melanoma primaries who have low disease burden micrometastases detected at SNB. The maximally selected rank (MSR) method^8^ was applied to DSS and DMFS separately. The method investigates all possible cut-points in the maximum SN metastatic deposit size and determines the cut-point that provides the best separation of the survival distribution (Kaplan–Meier curves) into two groups as the optimal threshold. Once an optimal cut-point was identified, univariable and multivariable Cox proportional hazard regressions were performed to evaluate whether the optimal cut-point remains significantly associated with outcomes after adjusting for potential confounding factors.

## Results

### Sentinel Node Disease Burden

We identified 1377 eligible patients in our database. The patients were stratified according to their primary tumor stage (namely pT1b-pT2a, pT2b-pT3a, pT3b-pT4a, and pT4b) to allow comparison with the current AJCC (8th edition) stage III classification system. Table 1 demonstrates the distribution of the risk factors analyzed across these four subgroups and the whole cohort. The incidence of SN+ was 17.3% (238/1377). Logistic regression analysis demonstrated that age, Breslow thickness, ulceration status, and primary tumor site were significant independent predictors of SN status (data not shown). The incidence of AJCC N1a, N2a, and N3a disease was 12.1% (166/1377), 4.8% (66/1377), and 0.4% (6/1377), respectively.

**Table 1:** Cross table of cohort stratified by primary tumor AJCC T-Stage (8th edition)

AJCC T stage		*N*	pT1b-pT2a [*n* = 776]	pT2b-pT3a [*n* = 299]	pT3b-pT4a [*n* = 190]	pT4b [*n* = 112]	Test statistic
Age	Median (IQR)	1377	61.0 (49.0–69.0)	66.0 (57.0–72.8)	67.0 (55.0–74.0)	69.0 (61.0–75.0)	*H* = 71.16, *p* < 0.001^b^
Sex	Male	1377	387 (49.9)	163 (54.5)	112 (58.9)	71 (63.4)	*χ*^2^(3) = 10.86, *p* = 0.013^a^
Primary site	Head and neck	1377	101 (13.0)	48 (16.1)	38 (20.0)	21 (18.8)	*χ*^2^(9) = 19.05, *p* = 0.025^a^
	Torso		323 (41.6)	102 (34.1)	84 (44.2)	38 (33.9)	
	Upper limb		155 (20.0)	76 (25.4)	32 (16.8)	23 (20.5)	
	Lower limb		197 (25.4)	73 (24.4)	36 (18.9)	30 (26.8)	
Subtype	SSM	1377	638 (82.2)	197 (65.9)	93 (48.9)	45 (40.2)	*χ*^2^(12) = 230.53, *p* < 0.001^a^
	Nodular		44 (5.7)	61 (20.4)	69 (36.3)	48 (42.9)	
	Acral		6 (0.8)	4 (1.3)	4 (2.1)	9 (8.0)	
	Lentigo maligna		39 (5.0)	15 (5.0)	8 (4.2)	3 (2.7)	
	Other		49 (6.3)	22 (7.4)	16 (8.4)	7 (6.3)	
Breslow	Median (IQR)	1377	1.2 (0.9–1.5)	2.4 (2.1–3.0)	3.4 (2.7–4.7)	5.8 (4.8–7.0)	*H* = 3707.30, *p* < 0.001^b^
Ulceration	Present	1377	14 (1.8)	70 (23.4)	127 (66.8)	112 (100.0)	*χ*^2^(3) = 767.29, *p* < 0.001^a^
SN status	Positive	1377	83 (10.7)	55 (18.4)	55 (28.9)	45 (40.2)	*χ*^2^(3) = 82.96, *p *< 0.001^a^
N stage^c^	N0	1377	693 (89.3)	244 (81.6)	135 (71.1)	67 (59.8)	*χ*^2^(9) = 86.79, *p* < 0.001^a^
	N1		60 (7.7)	38 (12.7)	38 (2.0)	30 (26.8)	
	N2		23 (3.0)	15 (5.0)	15 (7.9)	13 (11.6)	
	N3		0 (0.0)	2 (0.7)	2 (1.1)	2 (1.8)	
AJCC III^c^	IIIA	238	83 (100.0)	0 (0.0)	0 (0.0)	0 (0.0)	*χ*^2^(9) = 468.79, *p* < 0.001^a^
	IIIB		0 (0.0)	53 (96.4)	0 (0.0)	0 (0.0)	
	IIIC		0 (0.0)	2 (3.6)	55 (100.0)	43 (95.6)	
	IIID		0 (0.0)	0 (0.0)	0 (0.0)	2 (4.4)	
Deposit size	Median (IQR)	238	0.8 (0.3–2.0)	1.3 (0.4–4.1)	1.6 (0.5–5.3)	4.0 (1.8–8.0)	*H* = 23.98, *p* < 0.001^b^
ECS	Present	238	7 (8.4)	6 (10.9)	10 (18.2)	2 (4.4)	*χ*^2^(3) = 5.59, *p* = 0.133^a^

Data are expressed as *n* (%) unless otherwise specified

*N* indicates the number of non-missing values

^a^Pearson (degrees of freedom)

^b^Kruskal–Wallis with 3 degrees of freedom

^c^AJCC 8th edition

*SSM* superficial spreading melanoma, *SN* sentinel node, *AJCC III* final AJCC stage after a positive SN biopsy, *ECS* extracapsular spread, *IQR* interquartile range, *AJCC* American Joint Committee on Cancer

For those patients with AJCC stage III disease, the incidence of IIIA, IIIB, IIIC, and IIID disease was 34.9% (83/238), 22.3% (53/238), 42.0% (100/238), and 0.8% (2/238), respectively. The median MTDS for the whole cohort was 1.5 mm (IQR 0.5–4.5 mm), and the size of the MTDS was significantly associated with primary tumor stage (*p* < 0.0001) [Table 1]. The incidence of ECS in the SN+ cohort was 10.5% (25/238). ECS status was not associated with Breslow thickness, ulceration status, or AJCC III stage, but was significantly associated with N stage (*p* < 0.001) and MTDS (*p* < 0.001) [Table 2].

**Table 2:** Cross table for extracapsular spread

Extracapsular spread status		*N*	Absent [*n* = 213]	Present [*n* = 25]	Test statistic
Age	Median (IQR)	238	62.0 (51.0–70.3)	67.0 (57.7–75.0)	*H* = 2.36, *p* = 0.1261^b^
Sex	Male	238	118 (55.4)	16 (64.0)	*χ*^2^(1) = 0.67, *p* = 0.041^a^
Primary site	Head and neck	238	26 (12.2)	2 (8.0)	*χ*^2^(3) = 0.88, *p* = 0.083^a^
	Torso		105 (49.3)	12 (48.0)	
	Upper limb		30 (14.1)	5 (20.0)	
	Lower limb		52 (24.4)	6 (24.0)	
Subtype	SSM	238	157 (73.7)	15 (60.0)	*χ*^2^(4) = 7.58, *p* = 0.108^a^
	Nodular		40 (18.8)	4 (16.0)	
	Acral		4 (1.9)	2 (8.0)	
	Lentigo maligna		3 (1.4)	1 (4.0)	
	Other		9 (4.2)	3 (12.0)	
Breslow	Median (IQR)	238	2.4 (1.6–4.1)	2.8 (1.7–3.7)	*H* = 0.01, *p* = 0.906^b^
Ulceration	Present	238	84 (39.4)	11 (44.0)	*χ*^2^(1) = 0.19, *p* = 0.66^a^
N stage^c^	N0	238	0 (0.0)	0 (0.0)	*χ*^2^(2) = 15.94, *p* < 0.001^a^
	N1		157 (73.7)	9 (36.0)	
	N2		52 (24.4)	14 (56.0)	
	N3		4 (1.9)	2 (8.0)	
Deposit size	Median (IQR)	238	1.2 (0.4–4.0)	7.5 (3.4–11.3)	*H* = 31.19, *p* < 0.001^b^

Data are expressed as *n* (%) unless otherwise specified

*N* indicates the number of non-missing values

^a^Pearson (degrees of freedom)

^b^Kruskal–Wallis with 1 degree of freedom

^c^AJCC 8th edition

*IQR* interquartile range, *SSM* superficial spreading melanoma, *AJCC* American Joint Committee on Cancer

### Survival

**Fig. 1:**
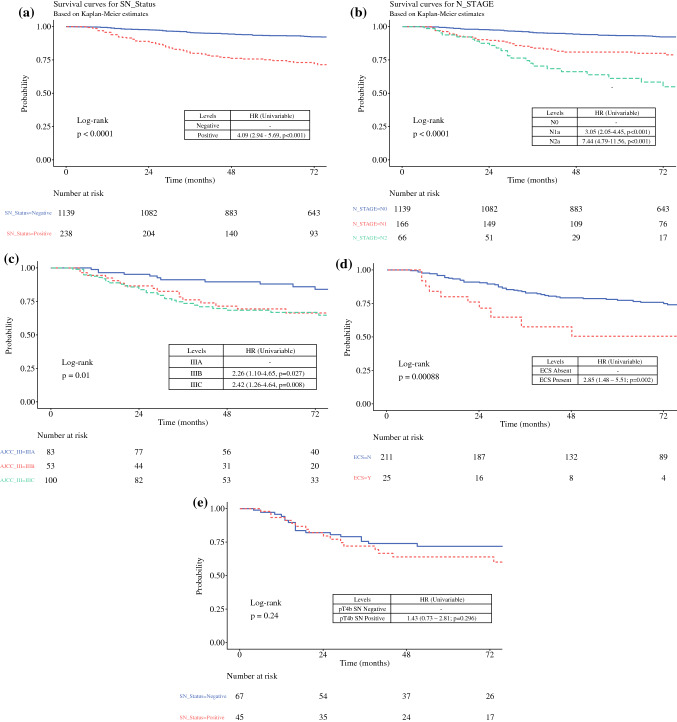
Kaplan–Meier curves showing disease-specific survival stratified by **a** SN status; **b** AJCC N stage (N3 subgroup not shown); **c** AJCC stage III subgroup (IIID subgroup not shown); **d** ECS status; and **e** SN status in the pT4b subgroup. *AJCC* American Joint Committee on Cancer, *ECS* extracapsular spread, *HR* hazard ratio, *SN* sentinel node

The median follow-up period was 75 months (IQR 47–111 months). An SN+ status was associated with worse DSS, with a 5-year survival of 74.5% compared with 93.4% for SN− patients (hazard ratio [HR] 4.09, 95% confidence interval [CI] 2.94–5.69; *p* < 0.001) [Fig. 1a]. Increasing AJCC N stage was also associated with worse DSS, with 5-year survival of 93.4% (95% CI 94.9–91.9%), 81.0% (95% CI 87.4–75.0%), and 61.2% (95% CI 76.2–49.1%) for stage N0, N1a, and N2a, respectively (N0 vs. N1: HR 3.05, 95% CI 2.05–4.54, *p* < 0.001; and N0 vs. N2: HR 7.46, 95% CI 4.8–11.59, *p* < 0.001) [Fig. 1b]. Comparison of the AJCC stage III curves demonstrated significantly worse DSS for the AJCC stage IIIB and IIIC groups compared with the AJCC stage IIIA group (IIIA vs. IIIB: HR 2.25, 95% CI 1.1–4.65, *p* = 0.027; and IIIA vs. IIIC: HR 2.42, 95% CI 1.25–4.64, *p* = 0.008), but pairwise comparison of the AJCC IIIB and IIIC groups showed no significant difference in DSS (*p* = 0.712) [Fig. 1c]. For the SN+ patients, the presence of ECS was associated with a significantly worse 5-year DSS (50.4% vs. 76.9%; HR 2.85, 95% CI 1.48–5.49; *p* = 0.002) [Fig. 1d]. Similar results were seen with analyses for DMFS and OS (electronic supplementary Figs. S1 and S2, respectively).

**Fig. 2:**
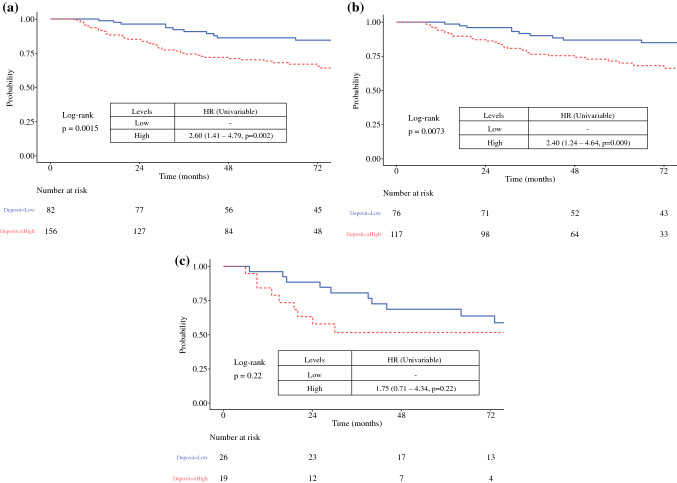
Kaplan–Meier curves showing disease-specific survival stratified by MTDS: **a** pT1b-pT4b cohort, and **b** pT1b-pT4a cohort, where low = MTDS ≤0.7 mm and high = MTDS >0.7 mm; **c** pT4b cohort, where low = MTDS ≤4.4 mm and high = MTDS >4.4 mm. *MTDS* maximum tumor deposit size, *HR* hazard ratio

The MSR method was used to identify an optimal MTDS cut-point across the whole cohort and for the pT subgroups used in the AJCC stage III classification table (8th edition). MSR analysis identified 0.7 mm as the optimum MTDS cut-point for the whole cohort, with 5-year DSS of 86.2% for the low-risk cohort and 68.1% for the high-risk cohort (HR 2.6, 95% CI 1.41–4.79; *p* = 0.002) [Fig. 2a]. Subgroup analyses showed that the optimal MTDS cut-point was 0.7 mm for the pT1b-pT2a, pT2b-pT3a, and pT3b-pT4a SN+ subgroups [Fig. 2b], but there was no significant optimal cut-point for the pT4b SN+ subgroup (MTDS cut-point = 4.4 mm; HR 1.75, 95% CI 0.71–4.34; *p* = 0.22) [Fig. 2c]. Further analysis demonstrated no difference in DSS between the SN+ and SN− subgroups for patients with pT4b primary melanomas (Fig. 1e]. For the SN+ subgroup with MTDS >0.7 mm, MSR identified 9 mm as the optimal cut-point, but this was not a significant predictor for DSS (*p* = 0.69).

**Fig. 3:**
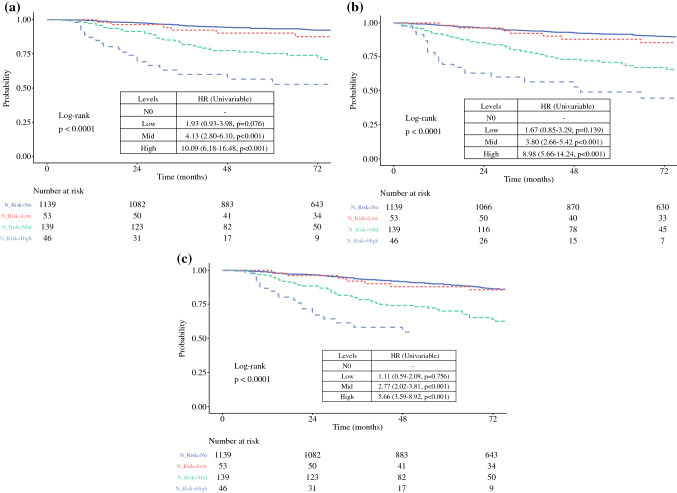
Kaplan–Meier curves stratified by the new nodal risk model: **a** disease-specific survival; **b** distant metastasis-free survival; and **c** overall survival. N0 = sentinel negative subgroup; low = MTDS ≤0.7 mm and no ECS; mid = MTDS >0.7 mm and no ECS; and high = ECS present (any MTDS). *MTDS* maximum tumor deposit size, *HR* hazard ratio, *ECS* extracapsular spread

A composite nodal risk score was developed using the risk factors of MTDS and ECS status according to the following criteria: low-risk: MTDS ≤0.7 mm and no ECS; intermediate-risk: MTDS >0.7 mm and no ECS; high-risk: any MTDS with ECS identified. Subanalysis showed that the intermediate- and high-risk groups were significantly associated with male sex, increasing Breslow thickness, and positive tumor ulceration status, whereas patients in the low-risk subgroup had a significantly lower incidence of nodal relapse (3.8%, 2/53; *p* < 0.001) [Table 3]. Figure 3a shows the resultant Kaplan–Meier survival curves. The 5-year DSS outcomes were 93.4%, 90.1%, 75.2%, and 52.6% for the SN− (pT1bN0-pT4bN0). low-risk, intermediate-risk, and high-risk subgroups, respectively (*p* < 0.0001). The pairwise comparisons between the subgroups were statistically significant except for the low-risk and SNB− between groups (HR 1.93, 95% CI 0.93–3.98; *p* = 0.076). Similar results were seen for DMFS and OS (Figs. 3b, c). Multivariate Cox regression analysis identified the new nodal risk model as a significant independent predictor of DSS when controlling for age, sex, primary, tumor site, ulceration status, and Breslow thickness (data not shown).

**Table 3:** Cross table stratified by new nodal risk model

New nodal risk score^d^		*N*	N0 [*n* = 1139]	Low [*n* = 53]	Mid [*n* = 139]	High [*n* = 46]	Test statistic
Age	Median (IQR)	1377	64.0 (52.0–71.0)	61.0 (49.7–69.3)	60.0 (49.2–69.0)	67.5 (60.9–74.1)	*H* = 0.28, *p* = 0.60^b^
Sex	Male	1377	599 (52.6)	16 (30.2)	91 (65.5)	27 (58.7)	*χ*^2^(3) = 20.40, *p* < 0.001^a^
Primary site	Head and neck	1377	180 (15.8)	5 (9.4)	20 (14.4)	3 (6.5)	*χ*^2^(9) = 29.83, *p* < 0.001^a^
	Torso		430 (37.8)	19 (35.8)	79 (56.8)	19 (41.3)	
	Upper limb		251 (22.0)	9 (17.0)	16 (11.5)	10 (21.7)	
	Lower limb		278 (24.4)	20 (37.7)	24 (17.3)	14 (30.4)	
Subtype	SSM	1377	801 (70.3)	42 (79.2)	106 (76.3)	24 (52.2)	*χ*^2^(12) = 25.68, *p* = 0.012^a^
	Nodular		178 (15.6)	7 (13.2)	22 (15.8)	15 (32.6)	
	Acral		17 (1.5)	1 (1.9)	2 (1.4)	3 (6.5)	
	Lentigo maligna		61 (5.4)	1 (1.9)	2 (1.4)	1 (2.2)	
	Other		82 (7.2)	2 (3.8)	7 (5.0)	3 (6.5)	
Breslow	Median (IQR)	1377	1.6 (1.1–2.5)	2.0 (1.5–2.7)	2.4 (1.6–4.0)	4.0 (2.4–5.8)	*H* = 90.61, *p* < 0.001^b^
Ulceration	Present	1377	228 (20.0)	10 (18.9)	54 (38.8)	31 (67.4)	*χ*^2^(3) = 75.92, *p* < 0.001^a^
Recurrence site	None	1377	974 (85.5)	35 (66.0)	82 (59.0)	17 (37.0)	*χ*^2^(9) = 157.32, *p* < 0.001^a^
	Local		44 (3.9)	10 (18.9)	15 (10.8)	3 (6.5)	
	Regional		28 (2.5)	2 (3.8)	17 (12.2)	9 (19.6)	
	Distant		93 (8.2)	6 (11.3)	25 (18.0)	17 (37.0)	
N stage^c^	N0	1377	1139 (100.0)	0 (0.0)	0 (0.0)	0 (0.0)	*χ*^2^(9) = 1559.91, *p* < 0.001^a^
	N1		0 (0.0)	43 (81.1)	100 (71.9)	23 (50.0)	
	N2		0 (0.0)	10 (18.9)	39 (28.1)	17 (37.0)	
	N3		0 (0.0)	0 (0.0)	0 (0.0)	6(13.0)	
AJCC III^c^	IIIA	238	–	27 (50.9)	49 (35.3)	7 (15.2)	*χ*^2^(6) = 34.44, *p* < 0.001^a^
	IIIB		–	16 (30.2)	31 (22.3)	6 (13.0)	
	IIIC		–	10 (18.9)	59 (42.4)	31 (67.4)	
	IIID		–	0 (0.0)	0 (0.0)	2 (4.3)	

Data are expressed as *n *(%) unless otherwise specified

*N* indicates the number of non-missing values

^a^Pearson (degrees of freedom)

^b^Kruskal–Wallis with 3 degrees of freedom

^c^AJCC 8th edition

^d^New nodal risk score is categorized as follows: low-risk = MTDS ≤0.7 mm/ECS absent; intermediate-risk = MTDS >0.7 mm/ECS absent; high-risk = ECS identified with any MTDS

*SSM* superficial spreading melanoma, *AJCC III* final AJCC stage after a positive sentinel node biopsy, *AJCC* American Joint Committee on Cancer, *IQR* interquartile range, *MTDS* maximum tumor deposit size, *ECS* extracapsular spread

Table 4 outlines the current risk factors for AJCC stage III melanoma and the incidence across the primary tumor subgroups and the whole cohort. According to the new nodal risk model, the incidence of low-risk disease was 22.3% (53/238) in the stage III cohort, 49% (26/53) in the pT2b-pT3a and pT3b-pT4a subgroups, and none in the pT4b subgroup. Conversely, 67.5% (56/83) of patients who mapped to the AJCC IIIA subgroup were restaged to either the intermediate- or high-risk subgroups.

**Table 4:** Cross table comparing current staging of sentinel node micrometastatic disease and the new nodal risk score, stratified by primary tumor AJCC stage, in accordance with the current AJCC stage III classification table

		*N*	pT1b-pT2a [*n* = 776]	pT2b-pT3a [*n* = 299]	pT3b-pT4a [*n* = 190]	pT4b [*n* = 112]	Test statistic
SN status	Positive	1377	83 (10.7)	55 (18.4)	55 (28.9)	45 (40.2)	*χ*^2^(9) = 82.96, *p* < 0.001^a^
Stage^b^	N0	1377	693 (89.3)	244 (81.6)	135 (71.1)	67 (59.8)	*χ*^2^(9) = 86.79, *p* < 0.001^a^
	N1		60 (7.7)	38 (12.7)	38 (20.0)	30 (26.8)	
	N2		23 (3.0)	15 (5.0)	15 (7.9)	13 (11.6)	
	N3		0 (0.0)	2 (0.7)	2 (1.1)	2 (1.8)	
AJCC III^b^	IIIA	238	83 (100.0)	0 (0.0)	0 (0.0)	0 (0.0)	*χ*^2^(9) = 468.79, *p* < 0.001^a^
	IIIB		0 (0.0)	53 (96.4)	0 (0.0)	0 (0.0)	
	IIIC		0 (0.0)	2 (3.6)	55 (100.0)	43 (95.6)	
	IIID		0 (0.0)	0 (0.0)	0 (0.0)	2 (4.4)	
New nodal risk score^c^	N0	1377	693 (89.3)	244 (81.6)	135 (71.1)	67 (59.8)	*χ*^2^(9) = 155.90, *p* < 0.001^a^
	Low		27 (3.5)	16 (5.4)	10 (5.3)	0 (0.0)	
	Mid		49 (6.3)	31 (10.4)	35 (18.4)	24 (21.4)	
	High		7 (0.9)	8 (2.7)	10 (5.3)	21 (18.8)	

Data are expressed as *n (%)*

*N* indicates the number of non-missing values

^a^Pearson (degrees of freedom)

^b^AJCC 8th edition

^c^New nodal risk score is categorized as follows: low-risk = MTDS ≤0.7 mm/ECS absent; intermediate-risk = MTDS >0.7 mm/ECS absent; high-risk = ECS identified with any MTDS

*SN* sentinel node, *AJCC III* final AJCC stage after a positive SN biopsy, *AJCC* American Joint Committee on Cancer, *MTDS* maximum tumor deposit size, *ECS* extracapsular spread

## Discussion

The latest AJCC classification system continues to confirm that the staging information obtained from an SNB remains pivotal in the decision-making process for the management of cutaneous melanoma at the initial diagnostic phase. The results of the MSLT-2 and DeCOG studies mean that the primary rationale for offering SNB has altered to identifying micrometastatic stage III patients who may benefit from adjuvant systemic therapy, rather than from surgery to the regional lymph nodes.^3,9–11^ Several prospective randomized controlled trials have demonstrated a recurrence-free survival benefit of adjuvant systemic therapy for patients with AJCC stage III metastatic melanoma,^12–15^ and most national clinical guideline committees now recommend it for high-risk resected stage III disease.^5,6^ The main rationale for offering adjuvant systemic therapy is based on the risk of recurrence and/or death from melanoma, but the risk of both short- and long-term toxicity from the treatment potentially outweighs the potential benefits for some. Therefore, the patient needs to have good clinical performance status and requires careful counseling in this scenario. The latest National Comprehensive Cancer Network (NCCN) guidelines^6^ state that stage IIIA melanoma patients (those with a pT1b-pT2a primary and a positive SNB) are eligible for adjuvant systemic therapy, although the phase III clinical trials to date have generally only included those with an MTDS >1 mm or those with ulcerated primaries in this subgroup.^16–18^ In contrast, those patients with AJCC stage IIIB–IIIC disease, who comprise virtually all of the patients with pT2b-pT4b melanomas with a positive SNB, are all eligible for adjuvant systemic therapy, according to national treatment guidelines, with treatment decisions based on concomitant comorbidities rather than according to nodal disease burden.^6^ There is genuine concern among the surgical oncology community that data from the landmark MSLT-1 study^2^ have rapidly been sidelined, particularly data that were highly suggestive of a subgroup of patients whose SNB was not only diagnostic but therapeutic.

With this latter point specifically in mind, we have undertaken an analysis assessing the outcome of SNB for patients with AJCC pT1b-pT4b primary cutaneous melanoma. The analysis focused on the burden of SN+ micrometastatic disease across the primary tumor subgroups and the DSS endpoint, in line with the current AJCC classification of stage III melanoma.^1^ Initial analysis of our data would suggest that the AJCC classification system has significant limitations for the stratification of micrometastatic melanoma staged with SNB. Table 3 highlights that 100% of the pT1b-pT2a and pT3b-pT4a primaries with a positive SNB map to the AJCC IIIA and IIIC subgroups, respectively, and, similarly, >95% of the pT2b-pT3a and pT4b primaries map to the AJCC IIIB and IIIC subgroups, respectively. These results occur regardless of the nodal disease burden, yet our data show a highly significant difference in DSS between the N1 and N2 disease subgroups. This finding is not new^19,20^ but is highly suggestive of the prognostic relevance of nodal disease burden, even in the micrometastatic scenario. However, the current N stage is skewed, being limited by the number of lymph nodes removed at the time of SNB (for instance, it is not possible to be staged N2a unless at least two nodes are sampled) and our data confirm that the low incidence of the N3a (any T stage with more than three positive SNs) and AJCC IIID (pT4bN3a) subgroups mean that these are largely irrelevant when discussing stage III micrometastatic disease (Table 3). Furthermore, the ratio of N1a to N2a disease remains constant across the pT subgroups.

Figure 1c highlights that no significant difference in DSS was identified between the AJCC IIIB and IIIC subgroups. On a superficial level, these data are not consistent with the current AJCC classification data;^1^ however, these two subgroups in that analysis included heterogeneous cohorts of patients with palpable nodal disease, microsatellites and in-transit metastases, which is the likely explanation for the discrepancy. In a more relevant comparison, a subanalysis of the dataset utilized for the current AJCC classification system demonstrated similar DSSs for MTDS from 0.2 to 2 mm, which included micrometastases from primary melanomas of all Breslow thicknesses. We performed a subgroup analysis according to the primary tumor stage and found that the consistent optimal cut-point for MTDS was 0.7 mm for SNB+ melanomas ranging from stage pT1b to pT4a. Importantly, we found that the low-risk (≤0.7 mm MTDS, no ECS) cohort had similar DSSs as their SNB− counterparts in the same pT-stage subgroup. This MTDS cut-point is also consistent with previous studies investigating models to predict non-SN involvement at CLND, in particular the validated N-SNORE system.^21,22^ We also highlight that the incidence of nodal relapse in our low-risk nodal cohort was very low (3.8%, 2/53), comparable with the SNB− cohort (2.6%, 28/1139) [Table 3]. We hypothesize that there may have been a potential therapeutic benefit from the SNB for these patients, both in terms of regional control and DSS, possibly derived from the early resection of the isolated metastatic focus in the SN(s). While we acknowledge that this hypothesis remains controversial, the data are nonetheless consistent with the outcome of the MSLT-1, MSLT-2, and DeCOG studies.^2,3,9,10^

Previous studies have shown a significant difference in DSS between SNB+ patients with or without ECS,^23^ in addition to a significantly increased risk of non-SN metastasis and regional relapse. Our analyses are consistent with these findings (Fig. 1d and Table 3). The data show that ECS was a significant independent predictor of distant relapse and death from melanoma, regardless of MTDS. We previously highlighted the term ‘nodal ulceration’ for ECS due to its phenotypical similarity of destroying the constraining adjacent epithelium and its similarity for upstaging the patient when identified, compared with primary tumor ulceration. Similarly, where Breslow thickness can be thought of as a surrogate marker for tumor burden of the primary, MTDS can be thought of as a similar surrogate marker for nodal tumor burden (i.e., a ‘nodal Breslow thickness’). Accordingly, we suggest that our composite nodal risk model is strengthened by utilizing similar risk factors that have been extensively validated for the primary tumor staging.^1^

The Keynote-716 study, a phase III trial investigating the efficacy of adjuvant pembrolizumab for resected stage II melanoma, recently reported the results of its interim analysis at 2 years.^24^ The analysis found a significantly improved survival for melanoma recurrence or death in the pembrolizumab arm compared with placebo (HR 0.65, 95% CI 0.46–0.92; *p* = 0.0066). The study has yet to report on the DSS endpoint but the results have prompted the FDA to approve the use of adjuvant pembrolizumab for resected stage II melanoma.^25^ Accordingly, the current debate in surgical oncology has shifted to reappraising those who are least likely to benefit from being staged by SNB, particularly if the likely subsequent management of the SNB+ group is no different from that of the SNB− group.^4,26^ Our data would indicate that the additional information afforded by the SNB for pT4b melanomas is limited, with no low-risk nodal micrometastatic disease in the SNB+ group and no difference in DSS between the SNB− and SNB+ subgroups. These data suggest that the same factors that are driving the advanced progression of the primary tumor are contributing to a similar rate of progression of the nodal metastasis, and it is becoming increasingly difficult to justify SNB for this very high-risk subgroup.

### Limitations

We readily acknowledge that our model has been generated from a cohort treated at a single-center and such a model would need to be extensively validated prior to adopting for routine clinical decision making. For example, larger analyses have demonstrated a significant survival difference between SN− and SN+ pT4b patients,^2, 27,28^ unlike the findings from our current study (Fig. 2c), indicating caution when interpreting the results of the analysis for this relatively small sample size (112/1377, 8.1%). Additional risk factors, including Dewar’s classification^29,30^ and non-SN status from patients who underwent CLND, may have modified our conclusions. Similarly, we acknowledge that the concept of MTDS as a prognostic biomarker has been described on multiple occasions,^31,32^ although we suggest that this analysis is the first to stratify MTDS according to pT stage in line with the latest iteration of the AJCC classification system for stage III disease.

## Conclusion

We propose a more granular classification system, based on two readily identified characteristics of SN metastasis. Tumor burden (MTDS) and nodal ulceration (ECS) status in the SN identifies not only low-risk patients in the AJCC IIIB and IIIC subgroups who may otherwise be observed but also high-risk patients in the AJCC IIIA subgroup who may benefit from adjuvant systemic therapy. pT4b melanoma primaries are very high risk and we suggest that staging this subgroup with SNB may no longer be justifiable.

## Supplementary Information

Below is the link to the electronic supplementary material.

Supplementary file1 (DOCX 1280 kb)
