# Supplementary material for: Risk Stratification of Sentinel Node Metastasis Disease Burden and Phenotype in Stage III Melanoma Patients
Source: Ann Surg Oncol. 2022 Nov 29;30(3):1808–19. doi: 10.1245/s10434-022-12804-6 (PMC9908720; doi:10.1245/s10434-022-12804-6)

**Supplementary Figure S1:** Kaplan-Meier curves showing distant metastasis-free survival stratified by a) sentinel node status; b) AJCC N-Stage (N3 subgroup not shown); c) AJCC Stage III subgroup (IIID subgroup not shown); d) ECS status; e) sentinel node status in the pT4b subgroup


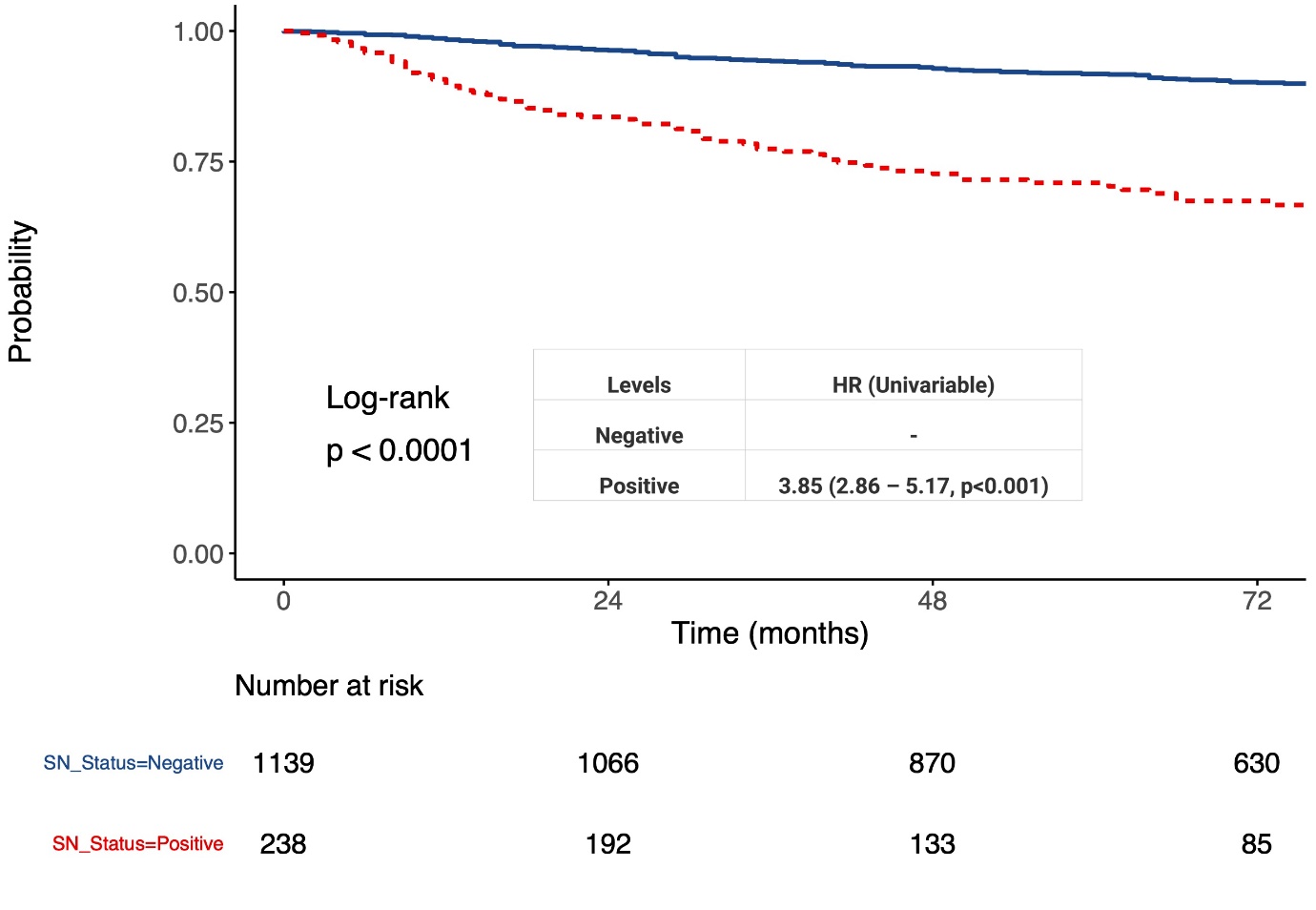


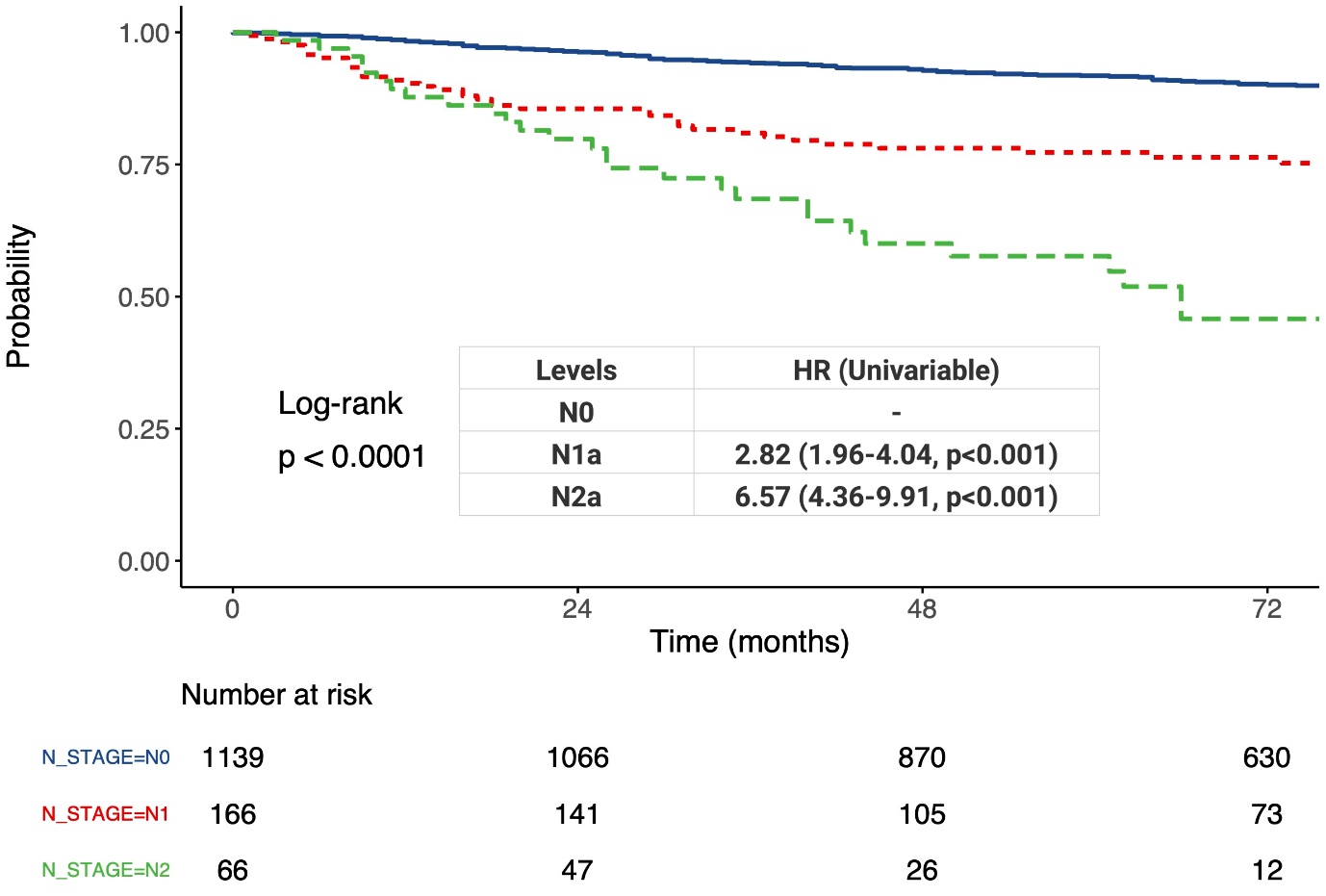


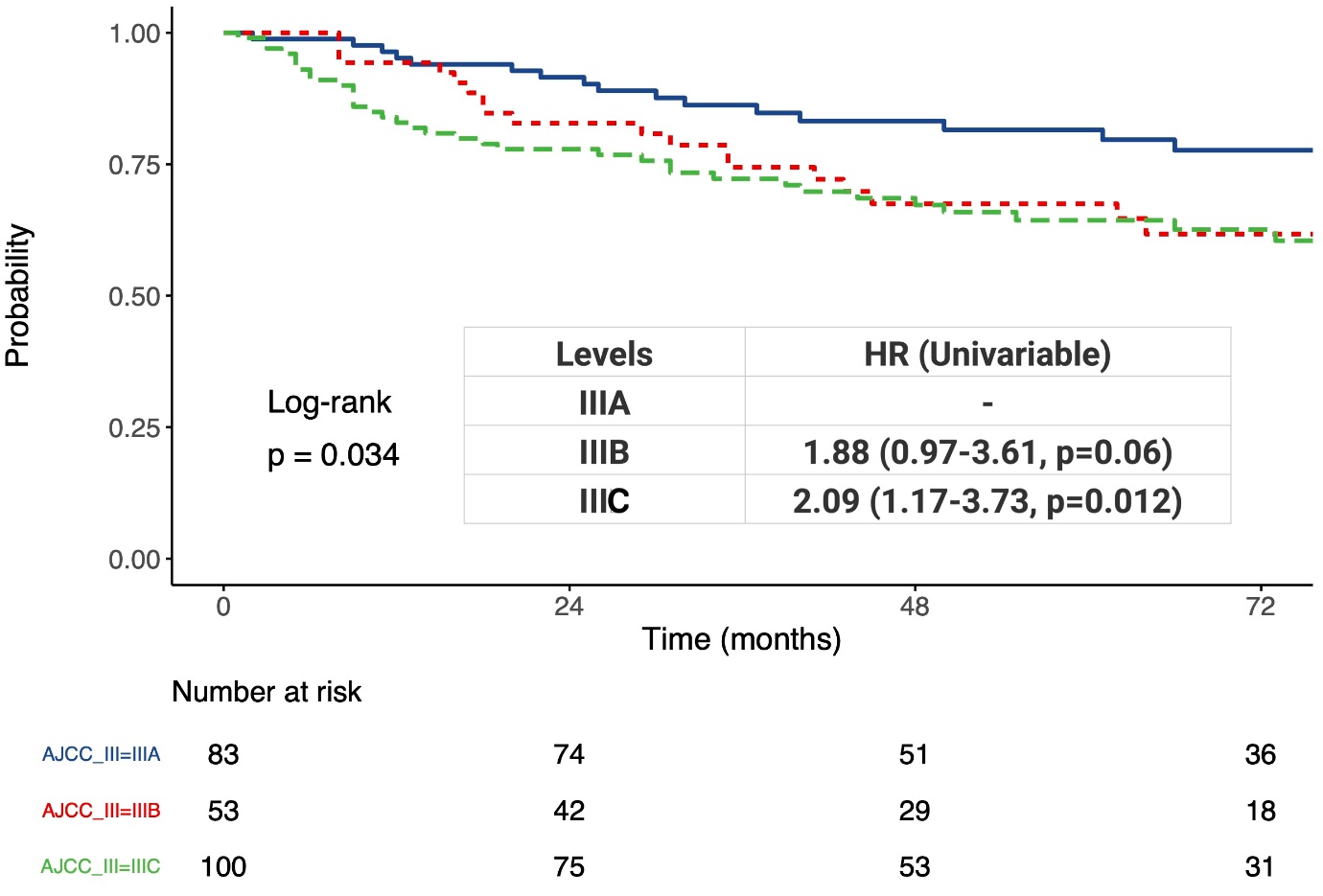


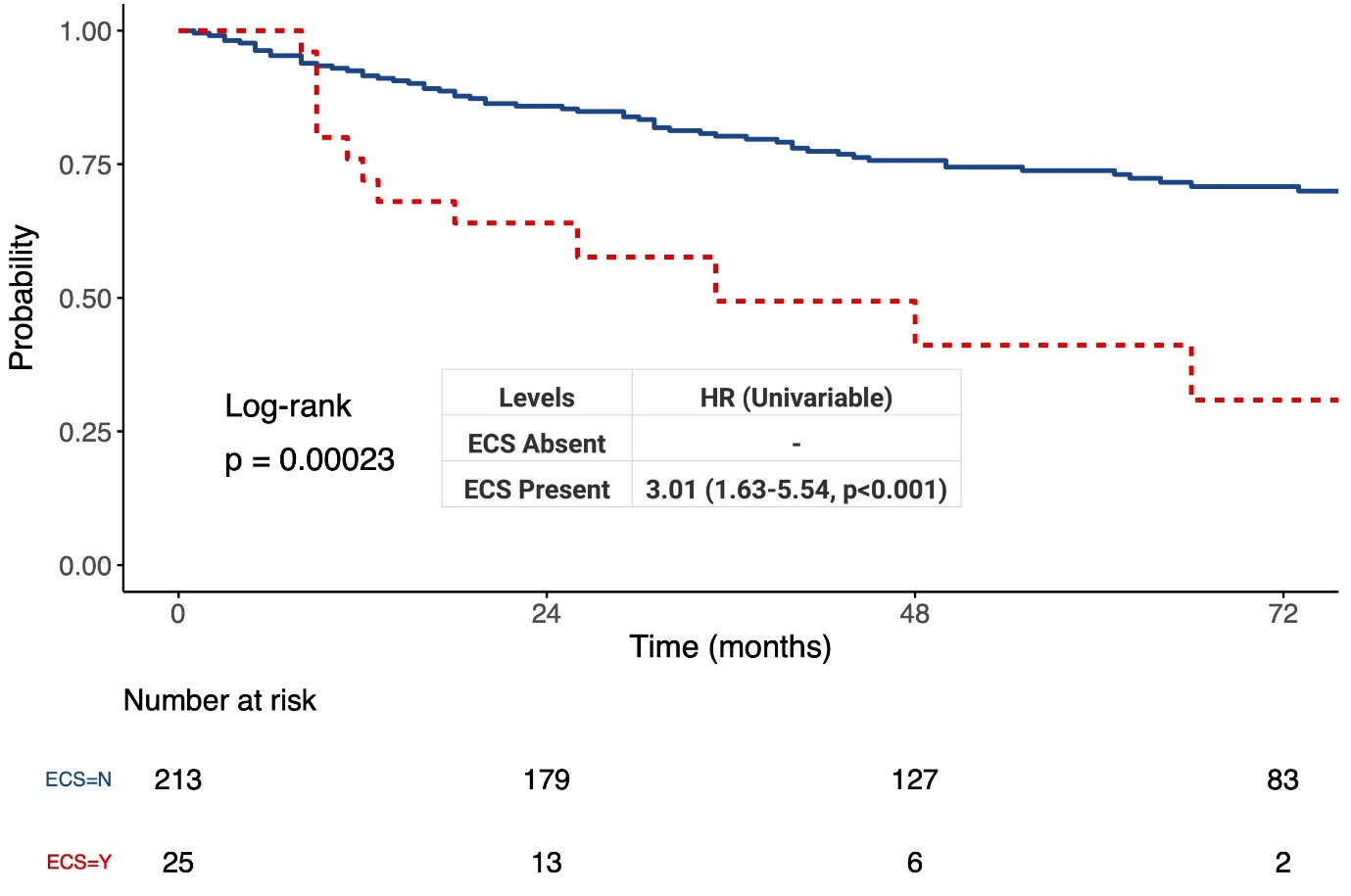


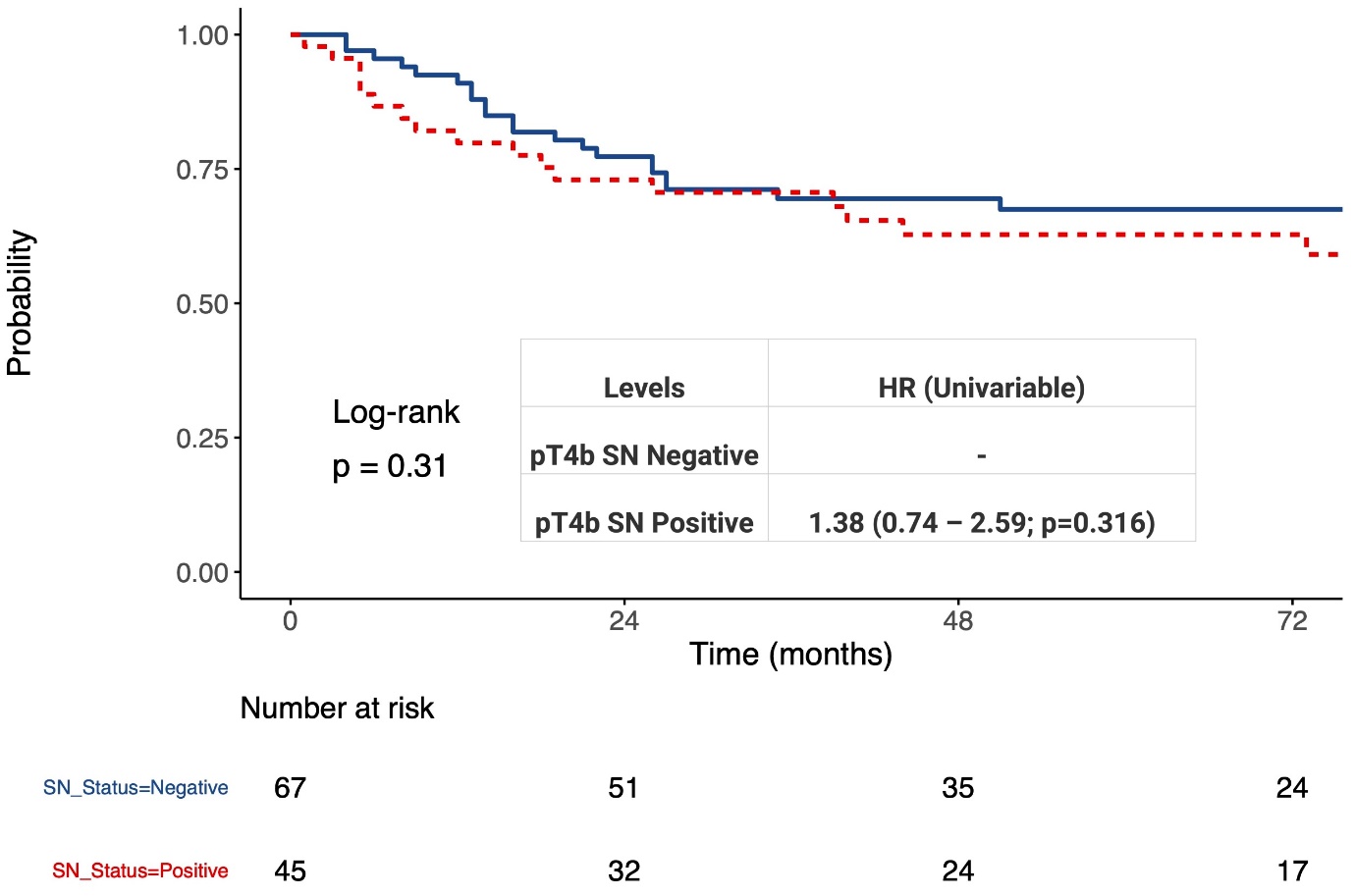


**Supplementary Figure S2:** Kaplan-Meier curves showing overall survival stratified by a) sentinel node status; b) AJCC N-Stage (N3 subgroup not shown); c) AJCC Stage III subgroup (IIID subgroup not shown); d) ECS status; e) sentinel node status in the pT4b subgroup


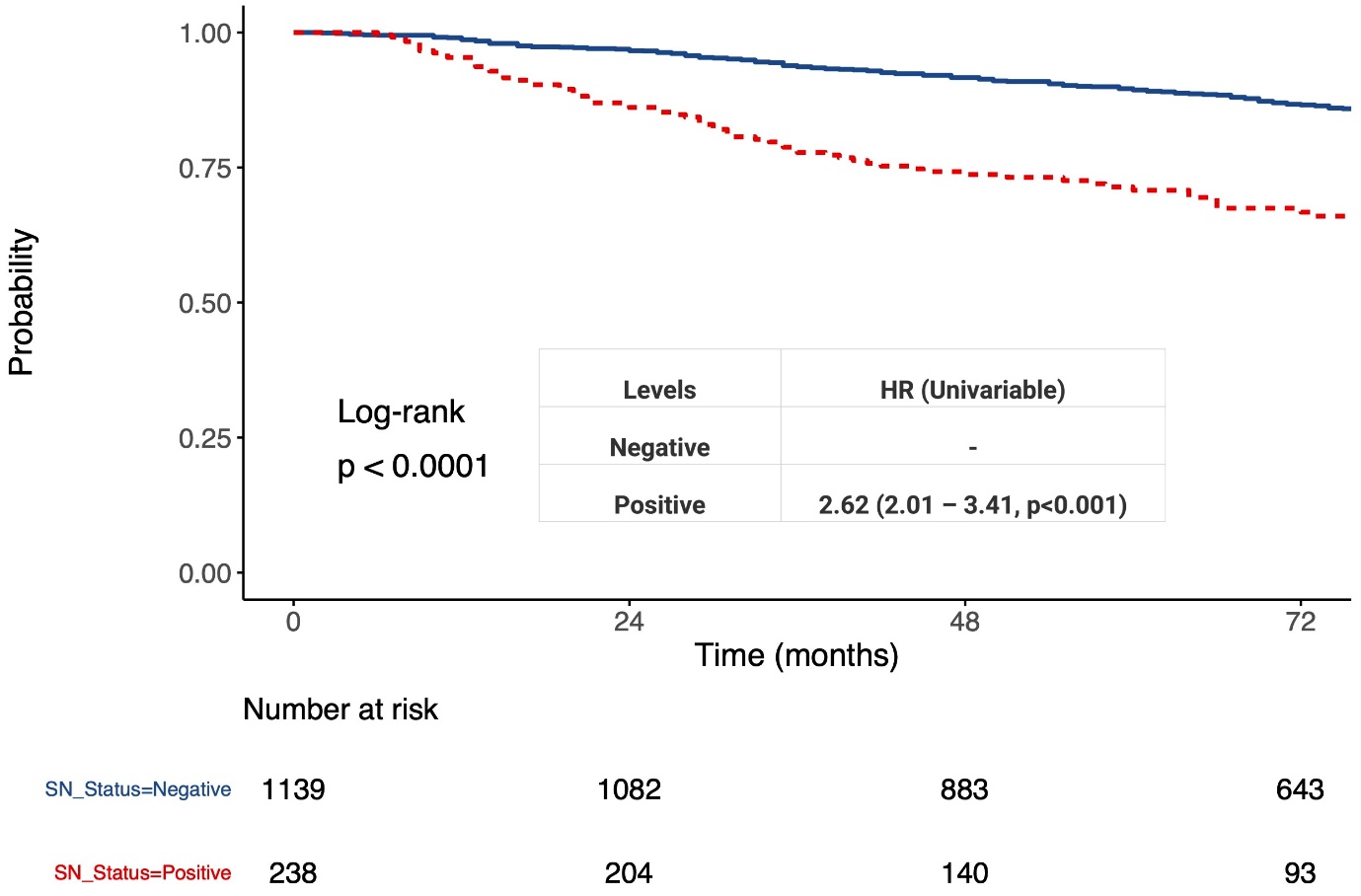


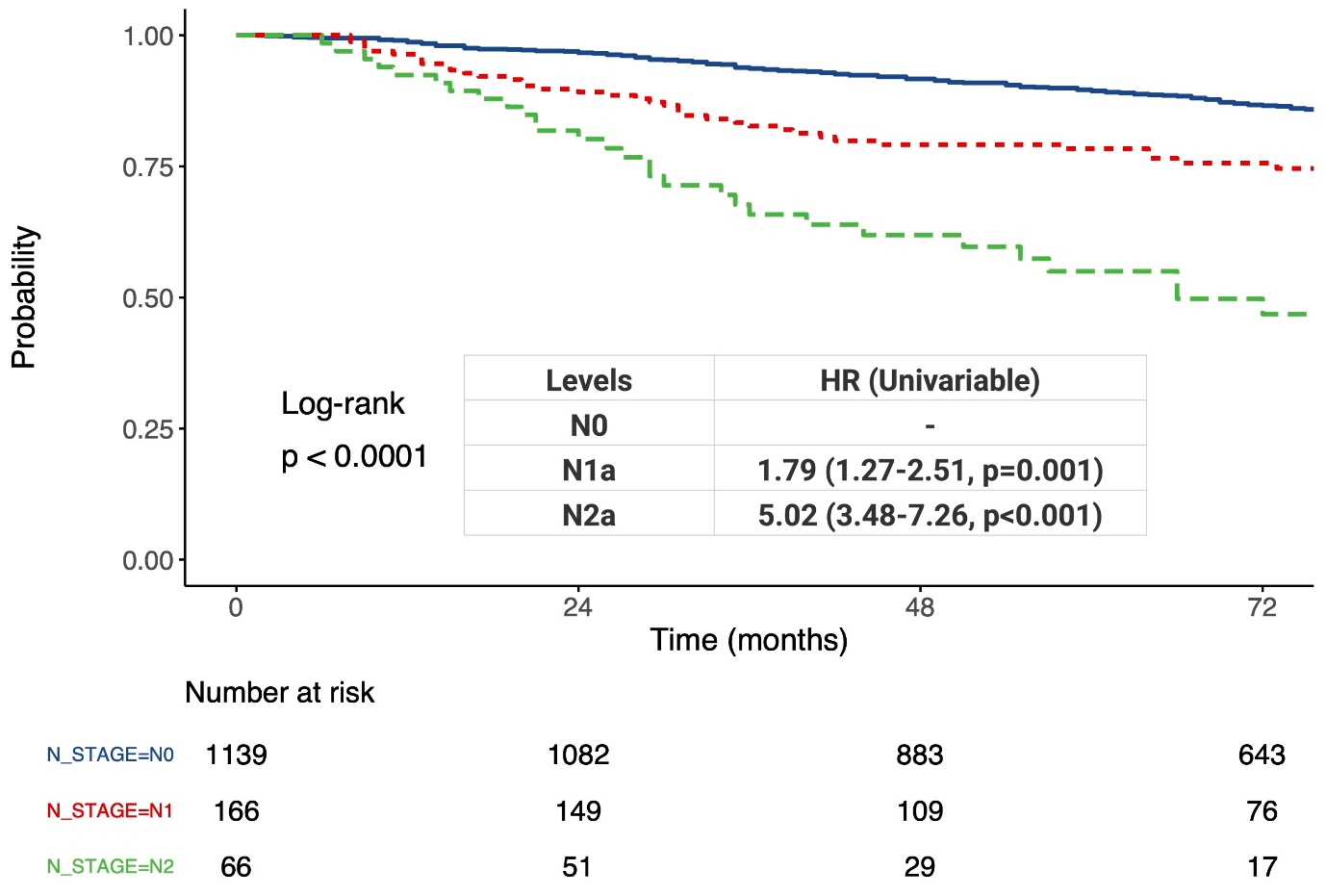


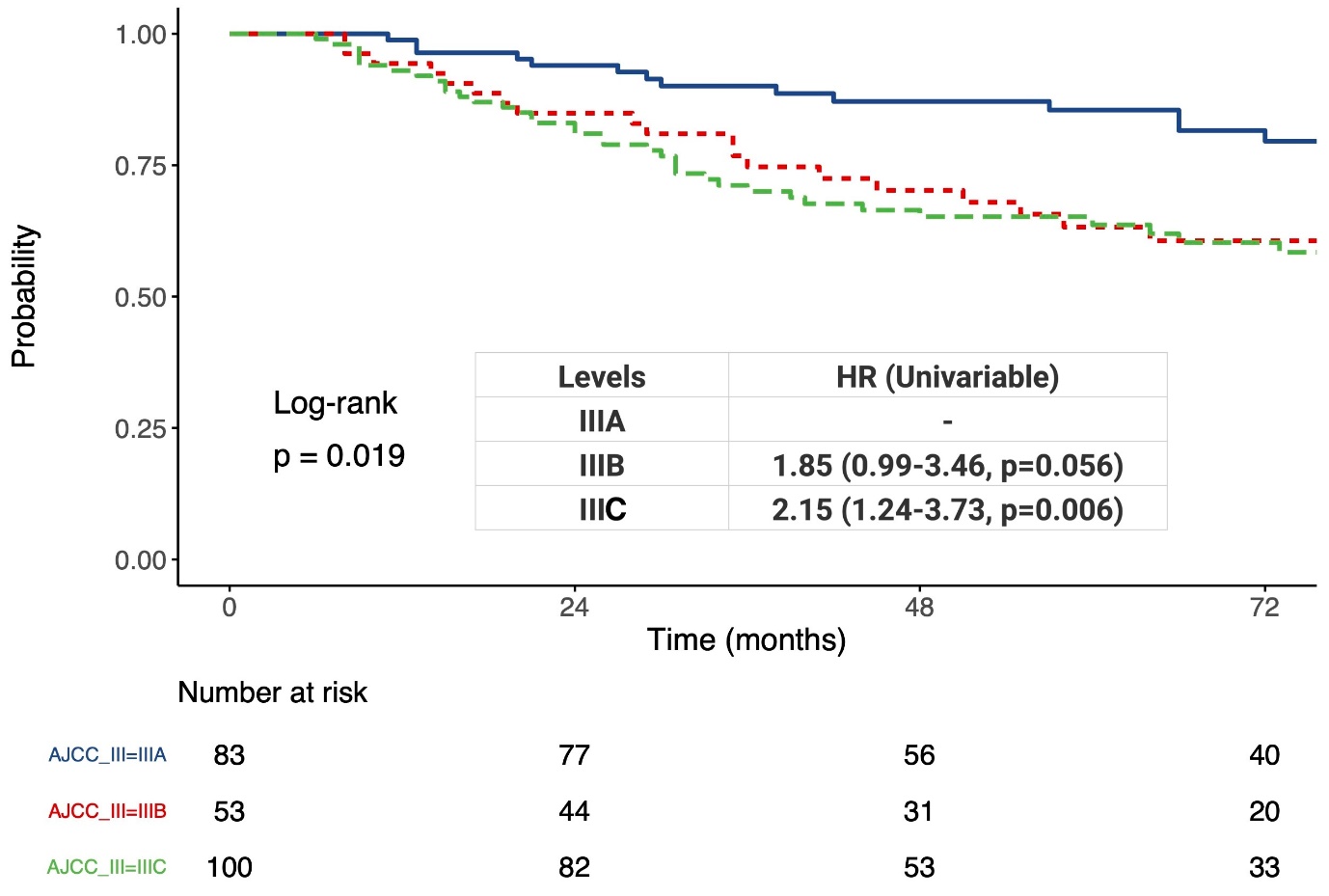


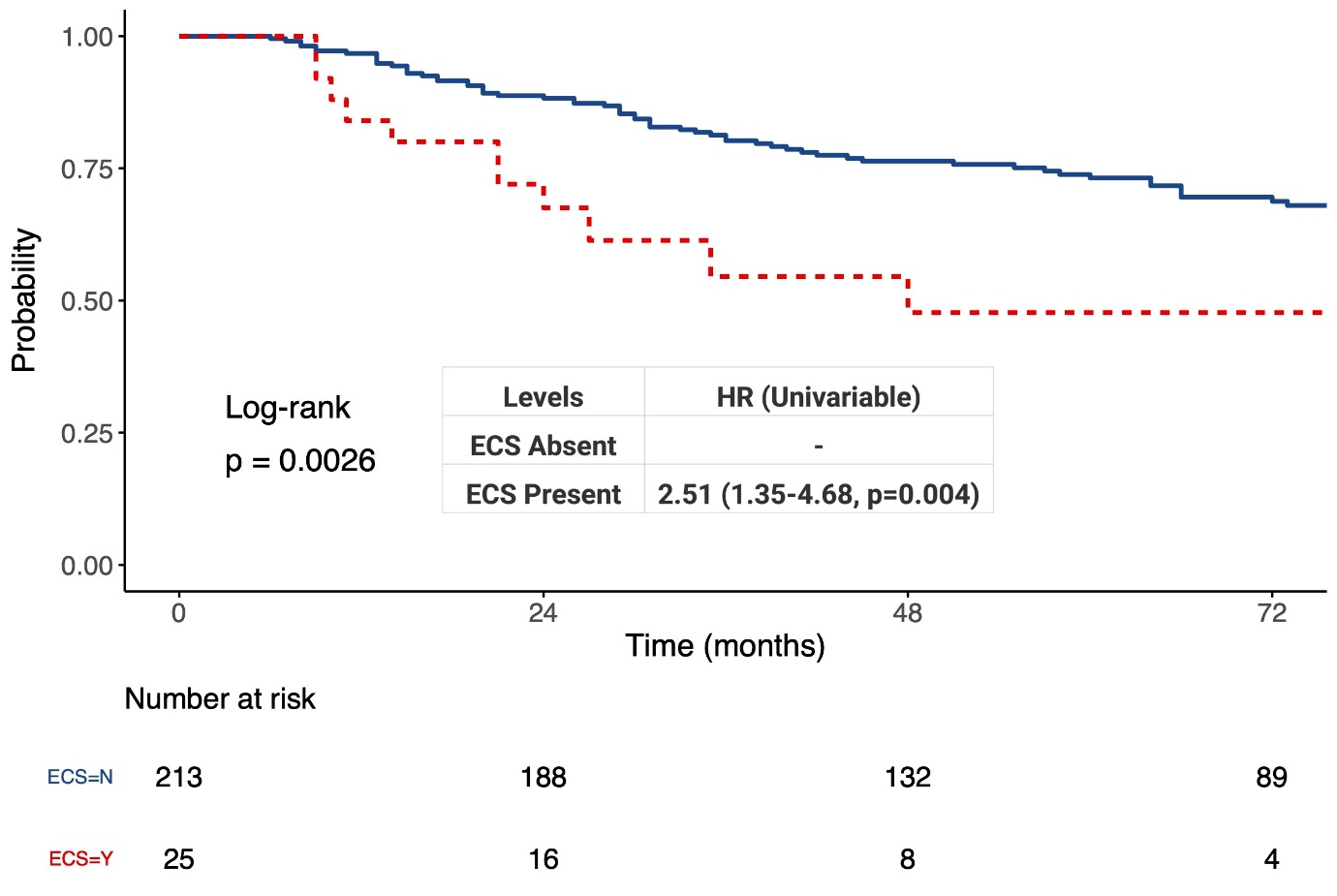


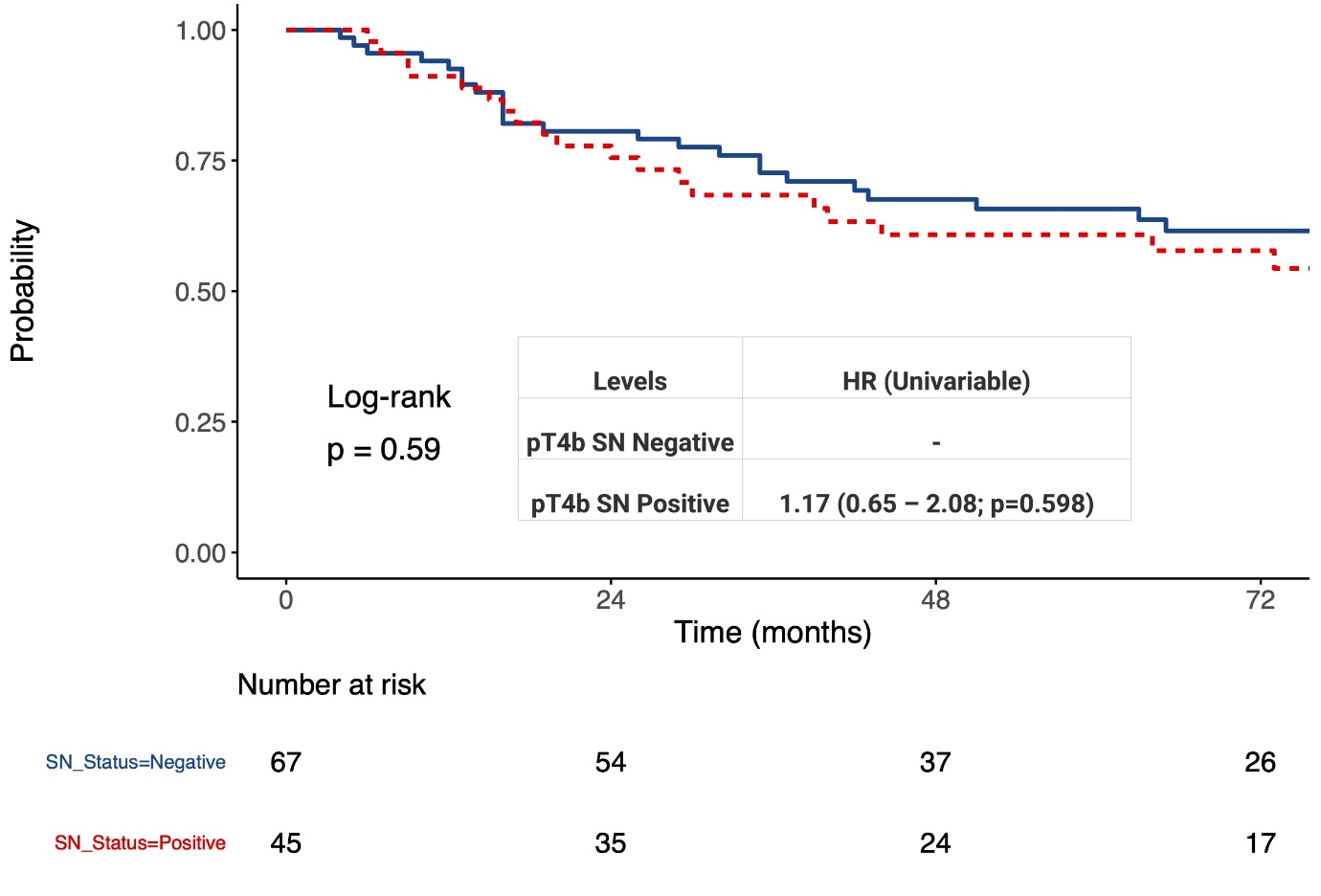

Supplement: Supplementary file 1 — Supplementary file1 (DOCX 1280 kb) [file 10434_2022_12804_MOESM1_ESM.docx]
